# Oncolytic Adenovirus in Cancer Immunotherapy

**DOI:** 10.3390/cancers12113354

**Published:** 2020-11-13

**Authors:** Malin Peter, Florian Kühnel

**Affiliations:** Department of Gastroenterology, Hepatology, and Endocrinology, Hannover Medical School, 30625 Hannover, Germany; peter.malin@mh-hannover.de

**Keywords:** oncolytic adenovirus, cancer immunotherapy, multi-stage, immunostimulatory, arming

## Abstract

**Simple Summary:**

Oncolytic adenoviruses are engineered to selectively replicate in and destroy cancer tissue. Moreover, these viruses are promising tools to restore antitumor immune response in cancer patients due to their high immunogenicity and the ability to interfere with the immunosuppressive tumor microenvironment. Due to these characteristics, oncolytic adenoviruses can activate tumors for already existing, systemic immunotherapies. The goal of this review is to provide an introduction into the common concepts of oncolytic adenoviruses, and to present their current status in clinical development. We also want to report in detail on strategies to optimize the immunoactivating properties of these agents for future application in multistage cancer immunotherapies.

**Abstract:**

Tumor-selective replicating “oncolytic” viruses are novel and promising tools for immunotherapy of cancer. However, despite their first success in clinical trials, previous experience suggests that currently used oncolytic virus monotherapies will not be effective enough to achieve complete tumor responses and long-term cure in a broad spectrum of cancers. Nevertheless, there are reasonable arguments that suggest advanced oncolytic viruses will play an essential role as enablers of multi-stage immunotherapies including established systemic immunotherapies. Oncolytic adenoviruses (oAds) display several features to meet this therapeutic need. oAds potently lyse infected tumor cells and induce a strong immunogenic cell death associated with tumor inflammation and induction of antitumor immune responses. Furthermore, established and versatile platforms of oAds exist, which are well suited for the incorporation of heterologous genes to optimally exploit and amplify the immunostimulatory effect of viral oncolysis. A considerable spectrum of functional genes has already been integrated in oAds to optimize particular aspects of immune stimulation including antigen presentation, T cell priming, engagement of additional effector functions, and interference with immunosuppression. These advanced concepts have the potential to play a promising future role as enablers of multi-stage immunotherapies involving adoptive cell transfer and systemic immunotherapies.

## 1. Introduction

Oncolytic viruses (OV) preferably replicate in and lyse tumor cells, and thus leave, healthy tissue unharmed. This common feature is either intrinsic or a consequence of genetic engineering [1]. OVs were initially designed to enable effective tumor cell lysis and virus spreading thereby ensuring a reliable control of viral replication in normal cells. However, it has been recognized that OVs exert multiple antitumor functions including the induction of innate and adaptive immune responses against the tumor. Oncolytic adenoviruses (oAds) have been among the earliest OVs to enter clinical trials. Onyx-015, an E1B55k mutant adenovirus for selective replication in p53 dysfunctional tumor cells, has been intensively investigated [2]. Though tumor responses had been observed in patients, particularly in combination with chemotherapy, the therapeutic efficacy did not meet the high expectations [3]. Nevertheless, these pioneering studies demonstrated that administration of oAds is well tolerated and safe. Additionally, lessons have been learned for the development of the next generation of viruses. Several advances in the fields of tumor immunotherapy have stimulated the interest in adenoviruses as oncolytic agents. First, there was the successful phase III study of the herpesvirus T-Vec and its subsequent approval by the U.S. food and drug administration (FDA), which delivered the final proof that OVs provide a clinical benefit for cancer patients [4]. There was also the growing perception that adaptive, tumor-directed immune responses are the essential therapeutic outcome of virotherapy [5]. A striking advance was the success of checkpoint inhibitors demonstrating that tumor immunotherapy even facilitates long-term cure [6]. However, the observation that the vast majority of cancer patients do not respond to these therapies opened up new future perspectives for the clinical application of OVs. The ability of oncolysis to induce tumor inflammation and to interfere with impaired immune functions in tumors suggests that OVs are promising agents to sensitize tumors for checkpoint inhibitors. Corresponding clinical studies are ongoing and are expected to deliver results soon [7]. Regarding the immunogenic properties of adenoviruses, oAds are presumably well qualified to meet this therapeutic need. Furthermore, oAds can be easily equipped with immunostimulatory transgenes to modulate the tumor microenvironment and to engage specific immune effector mechanisms. In this review, we want to give an overview on the currently existing platforms of oncolytic adenoviruses as well as the current state of their clinical development. We will report on current concepts of arming oAds with cytokines or alternative immune activators and will finally discuss the future prospects of oAds as an integrative part of multi-stage immunotherapies.

## 2. Adenovirus Cell Entry, Replication, and Immunogenicity

With more than 55 different serotypes, adenoviruses are ubiquitous pathogens that cause infections of the eyes, the respiratory or gastric tract with rather mild clinical manifestations in immunocompetent individuals [8]. Adenoviruses are non-enveloped, icosahedral viruses approximately 90 nm in size with a linear, non-integrating dsDNA genome ranging from 30–38 kb depending on the serotype. The proteins, which are expressed early during the viral replication cycle, exert regulatory functions including cell cycle induction, prevention of premature apoptosis, interference with pathogen defense, and escape from immune recognition. Typical late proteins are major capsid components such as hexon, penton, and fiber. Viral DNA replication and capsid assembly take place in the nucleus and infected cells undergo a lytic process to release the virus progeny. Since adenoviruses are able to infect a large spectrum of epithelial cells, they have been preferably adopted for gene transfer purposes and as oncolytic agents [9]. The commonly used serotype 5 infects cells by recognizing the coxsackievirus adenovirus receptor. After association with this primary receptor on the surface of a target cell, subsequent recognition of integrins by an RGD-motif, located in the capsid protein penton, initiates the endocytotic uptake of the viral particle.

Adenoviruses are highly immunogenic. Components of the virus capsid, the viral DNA, and specific intermediates expressed during the replication cycle are strong pathogen-associated molecular patterns (PAMPs) that are detected on all levels of cell entry. Their recognition by cellular pattern recognition receptors (PRR) triggers an inflammatory response comprising the release of numerous cytokines and chemokines (for review see [10]). In infected cells, oAds induce immunogenic cells death (ICD), an essential process for triggering adaptive antitumor immune responses and antitumoral memory. oAds kill infected tumor cells with features of necrosis/necroptosis, and autophagy [11,12,13] accompanied by release of high mobility group box-1 (HMGB-1), calreticulin, extracellular ATP, and heat shock protein 70 (Hsp70) [14,15,16]. Adenovirus-mediated ICD has been associated with induction of antitumor immune responses [17]. Consistently, it has been demonstrated by depletion of T cells in a Syrian hamster model that therapeutic efficacy of oAds was largely T-cell mediated [18]. In summary, these observations indicate that immunogenic cell death is an important prerequisite for therapeutic efficacy of oAds.

## 3. Current Concepts of Tumor-Selective Replicating Adenoviruses

The adenoviral E1 proteins drive infected cells into the S-Phase of the cell cycle and prevent premature apoptosis. These proteins have therefore been preferred targets of genetic manipulation to generate tumor-selective replicating adenoviruses. The adenovirus mutant dl-l520 (Onyx-015) lacks E1B-55k, a potent inhibitor of p53. As Onyx-015 is unable to degrade p53, it was originally assumed to productively replicate in p53 mutant tumor cells but not in cells with functional p53 [2]. However, subsequent studies have shown that replication in tumor cells was rather associated with other cellular functions such as late mRNA export [19,20]. A closely related oAd, the E1B-55k-deleted H101, has been approved for the treatment of head and neck cancer in China. Alternatively, p53-dysfunction has been addressed with oAds that exploit the virus-induced upregulation of p53 to activate mechanisms to suppress the expression of E1A and to inhibit the onset of adenovirus replication in normal cells [21,22]. Further mutant adenoviruses contain deletions in E1A, an essential protein for the onset of replication [23,24]. E1A binds to complexes containing E2F and the retinoblastoma protein (Rb) promoting the release of free E2F, which drives the cell to enter the cell cycle. Disruption of the Rb-binding site disables E1A to support adenoviral replication in resting cells without free E2F. Such E1A mutants are the basic modification of several oAds (referred to as dl922-947 or ∆24) and variants thereof are currently under clinical investigation [25]. Deletions in the N-terminus of E1A, responsible for binding to p300, further improve tumor-selective replication of the virus [26]. It is not well understood how these frequently used genetic modifications affect immunogenicity and immunogenic cell death. The sequence that is missing in Δ24-E1A is important for blocking the pathway of cyclic GMP-AMP synthase/stimulator of interferon genes (cGAS/STING) [27]. Proteins of the E3 region, also frequently deleted in oAds, are involved in regulation of adenoviral immune escape and nuclear factor kappa B (NFκB) activity levels [28,29]. It has been pursued to increase intrinsic immunogenicity, e.g., by inserting immunostimulatory CpG islands into the adenoviral backbone to enhance toll-like receptor (TLR)-9 signaling upon intracellular detection of the virus [30]. However, systematic studies on adenovirus immunogenicity in vivo are difficult, since syngeneic mouse models do not support productive replication of human adenovirus and may not reflect immunogenicity in the human system [31,32].

Targeted transcriptional control of E1A by tumor-specific promoters has also been a favored tool to generate oAds. Promoters for established tumor markers such as α-fetoprotein (AFP) or mucin-1 (MUC1) have been used for this purpose [33,34]. To broaden the spectrum of target cancers, promoters have been used that are activated by pan-cancer molecular alterations. Targeting tumor cells with a defective Rb-pathway, the E2F-1 promoter has been employed for control of E1A to facilitate tumor-selective replication [35,36]. As the hTert subunit of the human telomerase is expressed in 90% of human tumors, oAds have been generated containing E1A under control of the hTert-promoter [37,38]. Some of these viruses are subject of current clinical trials (see below). Additionally, artificial promoters have been developed to improve tumor selective E1A expression and virus replication. The oAd ICOVIR-7 has been provided with an insulated E2F promoter harboring additional E2F-responsive sites. The increased E2F-depency reduced systemic toxicity in immunocompetent mice [39]. The original hTert-promoter has been modified to contain further Sp1 and c-myc binding sites or a TATA-Box to increase effective replication in tumor cells [40,41]. Alternative strategies depend on aberrant expression of oncoproteins such as Y-box-binding protein 1 (YB-1) [42] or exploit hypoxic conditions in the tumor core by using a hypoxia-inducible factor 1α (HIF1α)-dependent promoter [43]. Recently, hybrid promoters containing hypoxia response elements (HRE) linked to either the E2F or hTert-promoter have been established to achieve potent viral replication in both hypoxic and normoxic regions of the tumor [44].

A further important aspect of tumor-selectivity is the ability to preferably recognize and infect cancer cells. Tumors have a tendency to downregulate the Ad5 primary receptor, which may impair adenoviral transduction of tumor tissue [45]. The capsid protein fiber, responsible for recognition of the coxsackievirus adenovirus receptor by adenoviruses, has therefore been a frequent target of genetic manipulations to improve tumor infection. One approach has been the introduction of an RGD-motif into the knob domain of fiber [46]. Circumventing the need of binding to the known primary receptors, this allows direct binding to cell surface integrins. More complex alterations comprise the exchange of the knob domain by the equivalent structures from alternative serotypes, such as Ad 3 or 35 [47,48]. These fibers bind to CD46 or desmoglein-2, which are not downregulated on the surface of tumor cells. Additionally, various strategies exist to redirect oncolytic adenoviruses to defined molecular targets on tumor cells (see review in [49]).

## 4. Translational Efforts and Clinical Development of Oncolytic Adenoviruses

Numerous clinical trials with oAds have been reported and several studies are currently ongoing. Without the claim of being exhaustive, Table 1 gives an overview of currently running clinical trials listed on https://clinicaltrials.gov. In the following section, some examples of oAds that have already entered clinical trials are described in more detail.

In preclinical studies, the telomerase-dependent OBP-301/telomelysin showed growth suppression in a panel of tumor cells and in xenograft models of lung cancer [38]. It was also demonstrated that oAds spread to the lymph nodes yielding an antimetastatic effect [50]. Safety of OBP-301 has been confirmed in phase I studies in various advanced solid tumors [51] and is currently being tested in phase II studies in metastatic melanoma, and in esophagogastric cancers in combination with pembrolizumab. It has been observed in bilateral, syngeneic models of colorectal and pancreatic cancer that a variant of OBP-301 in combination with an antibody targeting programmed cell death protein-1 (PD-1) yielded an abscopal effect on non-treated tumors confirming that oAds are promising agents to immunize tumors for checkpoint inhibitors [52].

DNX2401 (tasadenoturev), a delta24-RGD adenovirus, contains delta24-E1A to facilitate selective replication in Rb-dysfunctional cells. The ability to infect tumor cells is enhanced through the integration of an RGD-motif in the fiber. DNX-2401 treatment was effective in preclinical glioma models and showed immunoactivating properties in syngeneic pancreas tumors in mice [53,54]. Regarding therapeutic efficacy, Lang et al. reported that after a single intratumoral injection of DNX-2401 in glioma, 20% of patients survived more than three years, and also almost complete responses could be observed, resulting in a progression-free survival of more than three years [25]. Investigations on tumor specimens from patients receiving a neoadjuvant treatment suggest that DNX-2401 replicates and spreads within the tumor. Signs of effective immune activation, such as infiltration of CD8 T cells and T-bet+ cells, have been reported. DNX-2401 is now under clinical investigation with pembrolizumab in brain cancers. A further variant expressing OX40L (DNX-2440) has already been generated and subjected to clinical testing.

VCN-01 (Ad-E2F-Δ24RGD-PH20) is also an oAd for use in Rb-dysfunctional tumors. In this virus, Δ24-E1A is transcriptionally controlled by a promoter harboring E2F-1 responsive elements to boost replication in tumor cells through a positive feedback loop. The virus additionally expresses a hyaluronidase for improved virus spreading. VCN-01 has shown tumor selectivity in vitro, antitumoral effects in murine xenograft models, and increased spreading of virus infection [55,56]. VCN-01 effectively killed patient-derived retinoblastoma in vitro. Intravitreous administration in retinoblastoma xenografts led to tumor necrosis, improved ocular survival, and prevented dissemination. Data from a phase I trial showed the feasibility of vitreous administration and antitumor activity in vitreous seeds. Local inflammation of the retina has been observed, but no systemic complications occurred [57]. VCN-01 is currently involved in clinical studies in pancreatic cancer in combination with gemcitabine and nab-paclitaxel and also in head and neck cancer in combination with pembrolizumab.

CG0070 and ONCOS-102 are both selective for Rb-dysfunctional tumor cells and express granulocyte-macrophage colony-stimulating factor (GM-CSF). CG0070, an oAd5 that uses an E2F-responsive promoter for control of E1A, has been developed for application in non-muscle invasive bladder cancer (NMIBC) [58]. A phase II trial has shown an overall complete response rate of 47% at 6 months in patients with Bacillus Calmette–Guerin (BCG)-unresponsive NMIBC with acceptable toxicity [59]. ONCOS-102 is an Ad5/3 fiber chimeric oAd with favorable toxicity data in a phase I study [47]. Clinical studies of ONCOS-102 combined with chemotherapy in mesothelioma, with pembrolizumab in advanced melanoma, and together with a dendritic cell (DC)-vaccine for treatment of prostate cancer are ongoing. LoAd703 is an oAd5 containing chimeric fibers with an Ad35 knob and is additionally armed with the costimulatory factors CD40L and 4-1BBL. Intratumoral injection of LoAd703 inhibited tumor growth in a syngeneic pancreatic tumor model in mice, which could be further enhanced with gemcitabine [48]. LoAd infection promoted lymphocyte migration and stimulated DCs resulting in the activation of natural killer (NK) cells and the triggering of tumor-directed T cell responses. Currently, the safety and viroimmunotherapeutic activity of LoAd703 in combination with atezolizumab are being clinically investigated in several cancer entities including pancreatic cancer.

Enadenotucirev (or EnAd, formerly Colo-Ad1) has been generated by in vitro chimerization using adenovirus serotypes 3 and 11, and subsequent coevolution by serial passaging in colon cancer cells [60]. Safety of intravenous injections has been demonstrated in a phase I study [61]. An additional phase I study is being carried out in colorectal cancer patients prior to surgical removal and in combination with chemoradiotherapy. A further advancement is the EnAd-variant NG-641, expressing a fibroblast activation protein (FAP)-CD3 bispecific T-cell engager (BiTE), the chemokines chemokine ligand 9 (CXCL9) and CXCL10, and interferon alpha (IFNα), in an approach to attract and stimulate T cells to attack both tumor cells and cancer-associated fibroblasts (CAFs). This variant and also the variant NG-350A, which expresses a costimulatory, agonistic CD40 antibody, are in clinical testing.

## 5. Armed Oncolytic Adenoviruses

### 5.1. Arming with Transgenes to Amplify Tumor Lysis

Once the limitations of using first-generation oncolytic viruses as monotherapy became apparent, transgenes were introduced into existing oAd platforms with the intention to amplify tumor lysis and viral spread. When expressed in cells, the herpes simplex virus thymidine kinase (HSV-tk) gene converts a non-toxic prodrug ganciclovir (GCV) into a toxic agent, which is also distributed to neighboring cells to cause bystander cytotoxicity. Application of a E1B-55k-deleted oAd expressing HSV-tk and GCV improved survival of human colon carcinoma xenografts in mice [62]. Furthermore, oAds expressing HSV-tk and cytosine deaminase have been generated for the treatment of prostate cancer [63]. These oAds have also been used to deliver further cytotoxic or immunostimulatory payloads such as adenovirus death protein (ADP) or interleukin 12 (IL-12), respectively [64,65]. Ad5-yCD/mutTKSR39rep-mIL12, which expresses murine IL-12, improved local and metastatic tumor control in a preclinical prostate adenocarcinoma model accompanied by only mild local inflammation. The corresponding oAd expressing human IL-12 is being investigated in clinical trials.

The tumor necrosis factor- (TNF)-related apoptosis-inducing ligand (TRAIL) has been used in oAds with serotype 5/35 chimeric fibers. In vitro, Ad5/35.IR-E1A/TRAIL showed efficient virus spread and induction of apoptosis. Systemic administration eliminated preestablished liver metastasis in mice [66]. Fernández-Ulibarri et al. developed an oAd expressing a soluble RNase onconase fused to a tumor ligand (ONC_EGFR_). Upon internalization, the molecule induces tumor cell death through RNA degradation [67].

A critical aspect of arming with cytotoxic transgenes is the non-virus mediated cell killing, which may affect the productivity of viral infection [68]. It is also unclear how non-viral cell death affects the induction of tumor-directed immune responses.

Some monoclonal antibodies (mAb) against growth factor receptors are approved anticancer drugs. When systemically applied, these immunotherapies cannot exploit their full potential because of poor tumor penetration and side effects through normal tissue exposure. Taking advantage of a clinically established antibody against human epidermal growth factor receptor 2 (HER2), a full-length trastuzumab-expressing oAd has been constructed [69]. Ad5/3-Δ24-tras showed improved cytotoxicity in a panel of HER2 + cell lines and enhanced antitumor efficacy in a xenograft model of gastric cancer. Viral oncolysis by Ad5/3-Δ24-tras activated CD11c + DCs in lymph nodes in a NK cell-dependent manner. The Fc-terminus of the antibody also labels target cells for recognition by innate immune cells, which may induce antibody-dependent cell-mediated cytotoxicity (ADCC). This approach therefore combines direct antitumor activity and the engagement of additional immune effector mechanisms.

### 5.2. Arming with Matrix-Modifying Genes to Enhance Intratumoral Virus Spreading

Tumor cells are embedded in a dense network of extracellular matrix (ECM) and infection-resistant stroma cells, which impair effective distribution of the virus. To address this issue, oAds have been provided with matrix modifying genes such as TIMP2, TIMP3, MMP8, and relaxin. Expression of these matrix modifiers enhanced intratumoral viral spread and effectively inhibited tumor growth in cancer xenograft models in mice [70,71,72,73].

VCN-01, a clinically investigated oAd (see above), is armed with a soluble human sperm hyaluronidase (PH-20), which effectively degrades hyaluronan. Degradation of the ECM by PH-20 results in enhanced virus spreading in xenografted tumors [56]. Mutants of the proteoglycan decorin have been used to improve viral distribution and tumor penetration by oAds [74]. In the future, it will be interesting to see how degradation of the ECM can promote leukocyte infiltration and immune activation of the tumor microenvironment. Recently, it has been shown with a relaxin-expressing oAd that the ECM degradation enhanced tumor penetration by a systemically administered therapeutic antibody. When oAds additionally expressed IL-12 and granulocyte-macrophage colony-stimulating factor (GM-CSF), tumors were effectively converted into an immunoactivated state responsive to PD-1 checkpoint inhibition [75].

### 5.3. Arming with Antiangiogenic Transgenes

Angiogenesis is an important target of immunotherapies in clinical oncology. oAds have been armed with antiangiogenic mechanisms to enhance the antitumor effect of oncolysis. In human hepatocellular carcinoma (HCC) cells and in xenografts in mice, Li et al. showed anti-angiogenesis and antitumoral effects when endostatin was expressed by the E1B-55k deleted oAd CNHK200-mE [76]. Xiao and colleagues generated ZD55-VEGI-251, also an E1B55k-deleted oAd, armed with a secreted isoform of vascular endothelial cell growth inhibitor [77]. VEGI-251 inhibited angiogenesis in chick chorioallantoic membranes and suppressed tumor growth in xenograft models. Decorin, which is able to suppress multiple tyrosine kinase receptors including c-Met and the Wnt/β-catenin pathway, has also been employed. In a nude mice model of human prostate cancer, the decorin-expressing Ad.dcn reduced tumor burden, significantly inhibited skeletal metastases and improved survival [78]. The group of Chae-Ok Yun suppressed vascular endothelial growth factor (VEGF) by expressing VEGF-specific short-hairpin RNA (shRNA) or by expression of an artificial zinc-finger protein (F435-KOX) targeting the VEGF promoter [79,80].

### 5.4. Arming with Immunostimulatory Cytokines and Chemokines

Corresponding to the diversity of immune mechanisms that can be dysfunctional in tumors, various immunostimulatory transgenes have been integrated into oAds to stimulate effective antitumor immune responses. Since systemic administration of potent immunostimulatory factors, such as type I Interferons, tumor necrosis factor alpha (TNFα), or interleukin 12 (IL-12), may have considerable side effects, delivery by oAds provides an attractive option to focus cytokine activity on the target tumor.

In tumor cells, IFNs exert pleiotropic effects including the activation of the immune proteasome, the upregulation of major histocompatibility complex (MHC) class I and II, and potent activation of NK cells and cytotoxic T lymphocytes (CTLs). Shashkova et al. integrated IFNα into an oAd (KD3-IFN), which should render replication more sensitive to the IFNα response in normal cells [81]. The authors were able to confirm a decreased off-target toxicity in HCC xenografts in nude mice and in an immunocompetent model of kidney cancer in Syrian hamsters. A cyclooxygenase (Cox) 2-dependent oAd expressing IFNα was capable of inhibiting tumor growth in a Syrian hamster model of pancreatic cancer [82]. In an immunocompetent mouse model of Lewis lung carcinoma, co-application of an oAd in combination with a non-replicating Ad-IFNβ has been investigated [83]. This binary strategy prolonged interferon expression and improved antitumoral immune responses. Efficient delivery of a non-replicating transgenic adenovirus by coinfection with an oAd has been initially shown in an approach of cancer gene therapy [84]. Regarding armed virotherapeutic vectors, this binary approach is particularly promising for immunostimulatory transgenes. Assuming that enough events with single virus transduction will occur, the binary method holds promise to maintain cytokine expression beyond clearance of the oAd.

The potent antitumor functions of TNFα have been well known for decades. Loco-regional delivery of TNFα by oAds promises potent antitumor activity with limited side effects. Hirvinen et al. showed that the TNFα-armed Ad5/3-E2F-delta24 vector led to increased tumor destruction due to TNFα-mediated apoptosis, immunogenic cell death, and induction of antitumor immune responses, including tumor-antigen-specific T cells [85]. A corresponding virus with additional expression of interleukin 2 (IL-2) (Ad5/3-E2F-D24-hTNFα-IRES-hIL2 or TILT-123) is currently under clinical investigations. IL-2 is a central cytokine for survival and proliferation of T cells qualifying TILT-123 to augment the transfer of tumor infiltrating lymphocytes (TIL). In an immunocompetent Syrian hamster tumor model, concomitant transfer of TILs and virus application resulted in a 100% cure of treated animals [86]. The virus has also been used to support tumor infiltration with chimeric antigen receptor (CAR) transgenic T cells [87]. By using an ex vivo ovarian cancer (OVCA) model derived from patient samples, enhanced levels of proinflammatory signals (IFNγ, CXCL10, TNFα and IL-2) associated with a concomitant activation of CD4 and CD8 TILs could be observed when tumor cells were infected with TILT-123 [88]. In response to autologous, T cell-depleted OVCA cultures, which had been infected with TILT-123, TILs secreted high levels of IFNγ. These observations confirmed the use of TILT-123 in adoptive cell transfer.

Several oAds have been armed with IL-12, an essential cytokine involved in inflammation and proliferation of effector T cells and NK cells. Using the hypoxia-dependent Ad-DhscIL12 in a Syrian hamster model of pancreatic cancer, Bortolanza et al. showed active viral replication and enhanced transgene expression in vivo resulting in potent antitumor effects and less toxicity due to shorter systemic exposure [89]. Lee et al. investigated the oAd YKL-IL12/B7 expressing IL-12 and B/7-1 (CD80), a ligand of the costimulatory CD28 receptor, on T cells. In a syngeneic murine B16-F10 melanoma, the virus showed effective tumor growth inhibition including complete tumor regressions and improved survival [90]. Using the oncolytic Ad-TD-nsIL12, which expresses a non-secreted version of IL-12, Wang et al. were able to reduce off-target toxicity of IL-12 [91].

IL-24 is an immunomodulatory cytokine with profound antitumor effects through immune activation, induction of tumor cell apoptosis and inhibition of angiogenesis. IL-24-expressing oAds have shown antitumor efficacy in vitro and in xenografts in mice [92,93]. IL-4 has been used to promote intratumoral leukocyte infiltration [94]. The cytokine IL-18 induces IFNγ production through T cells and NK cells. Using the IL-18-armed ZD55 in xenograft models, Zheng et al. could observe stronger antitumor responses and inhibition of tumor angiogenesis [95]. Choi et al. generated oAd expressing IL-12 in combination with IL-18, or IL-23, respectively, and demonstrated enhanced antitumor efficacy in B16-F10 melanoma associated with an improved Th1/Th2 cytokine ratio and infiltration of NK and T cells [96,97]. Cytokines have also been combined with the chemokine CCL21, which binds to CCR7 on naïve T cells and DCs and promotes their attraction to the tumor [98,99].

Alternative options for immune arming are factors that directly target immunosuppression in the tumor microenvironment. Seth et al. have targeted transforming growth factor β (TGFβ) with a soluble TGFβ-receptor II protein fused to a human immunoglobulin (IgG) Fc fragment [100]. By using an oAd expressing sTGFβRII-Fc (rAd.sT), the authors showed in a xenograft mouse model tumor regression in 85% of treated animals. rAd.sT enhanced the efficacy of concomitant anti-PD-1 and anti-cytotoxic T-lymphocyte-associated protein 4 (CTLA-4) treatment in an immunocompetent 4T1 breast cancer model [101].

### 5.5. Immunological Arming to Improve Antigen Presentation

To enable successful tumor-directed T-cell immunity, effective presentation of tumor antigen by DCs needs to be restored. The chemokine GM-CSF promotes maturation and activation of antigen presenting DCs from myeloid precursors. oAds armed with GM-CSF have been used to elicit T-cell mediated antitumoral responses [58,102]. CG0070 is a GM-CSF-armed oncolytic Ad5 involved in clinical investigation as described above. Using Ad5-∆24-GMCSF, Cerullo et al. showed tumor-specific immunity in an immunocompetent syngeneic hamster model [102]. In 20 patients with advanced solid tumors, responses could be observed including two complete tumor responses. The administration of Ad5/3-∆24-GMCSF has been investigated in tumor patients showing a clinical benefit according to RECIST criteria in 8/12 radiologically evaluated individuals. The data revealed that oAd treatment affected immune responses specific for the tumor antigen survivin [103]. A correlation of antitumoral and antiviral immune responses has been confirmed by Kanerva et al. [104]. An oAd expressing both GM-CSF and IL-12 has been used to support the administration of a DC vaccine. Tumor infection with Ad-ΔB7/IL12/GMCSF promoted migration of DCs to tumor-draining lymph nodes [105]. However, GM-CSF also has protumorigenic and immunosuppressive functions by recruiting myeloid suppressor cells and impairing immune responses [106,107]. In pancreatic cancer, tumor-released GM-CSF supports the development of an immunosuppressive subset of DCs, which promotes metastasis [108]. Alternative options to improve intratumoral antigen presentation by oAds include the co-expression of Fms-like tyrosine kinase-3 ligand (Flt3L) and GM-CSF [109], or a combination of Flt3L with macrophage inflammatory protein 1α (MIP-1α, CCL3) [110].

### 5.6. Arming with Transgenes Addressing T Cell Costimulation or Immune Checkpoints

Pharmacological blockade of inhibitory immune checkpoints or activation of costimulatory receptors are potent strategies to activate antitumor T cells. Dias et al. used a full length anti-CTLA4 monoclonal antibody expressed by Ad5/3-Δ24-CTLA to combine oncolysis and checkpoint inhibition [111]. Intratumoral expression allowed high local levels of the checkpoint inhibitor. In patient-derived peripheral blood mononuclear cells (PBMCs), the authors observed T cell activation and αCTLA4-mediated apoptosis. The PD-1/PD-L1 is an inhibitory checkpoint regulating the activity of peripheral T cells. In prostate cancer models, Tanoue et al. showed that an oAd combined with a helper-Ad expressing a PD-L1-blocking mini-antibody supported the intratumoral activity of adoptively transferred CAR T cells [112]. The specific benefit of viral delivery was confirmed by the demonstration that local expression of the PD-L1-blocking minibody was superior compared with systemic infusion of αPD-L1 IgG. An equivalent approach using helper-dependent adenoviruses for expression of the PD-L1 blocking mini-antibody and IL-12-p70 for immune stimulation augmented the activity and persistence of CAR T cells in murine models of head and neck squamous cell carcinoma (HNSCC) [113].

CD40 is a costimulatory receptor expressed on antigen-presenting cells (APCs), mostly B cells, macrophages and DCs. Interaction of CD40 with its ligand CD40L induces cytokine production, increases MHC class II-dependent antigen presentation and thus supports the priming and expansion of T cells. Tumor infection with the CD40L-armed AdEHCD40L reduced the growth of xenografted human myeloma [114]. The oncolytic Ad5/3-hTERT-E1A-hCD40L (CGTG-401) induced multiple antitumor effects including reduced tumor growth via apoptosis, increased number of cytotoxic CD8 T cells in the tumor, and upregulation of T_H_1 associated cytokines [115]. Administration of CGTG-401 in nine patients with advanced solid tumors demonstrated that the treatment was well tolerated, and immunological responses could be confirmed [116]. The Hemminki group also recently showed that a CD40L-expressing oAd enabled effective antitumoral DC-therapies in humanized mice [117].

APCs express the co-stimulatory molecule 4-1BB ligand (4-1BBL), and 4-1BB antibodies are known to stimulate potent antitumor immune responses. The oncolytic adenovirus LoAd703, armed with 4-1BBL together with a trimerized CD40L, is currently being tested in clinical trials as described above [48]. A further approach studying the combined expression of 4-1BBL and IL-12 (Ad-ΔB7-IL12/4-1BBL) demonstrated a synergistic enhancement of IFNγ levels compared to single cytokine viruses and supported the administration of DCs through an enhanced T_H_1-mediated antitumor immune response [118].

The costimulatory OX40 ligand (OX40L) binds to OX40 on T cells and promotes T cell activation. Application of the OX40L-expressing oAd Delta-24-RGDOX showed intratumoral activation of lymphocytes and the development of a tumor-specific CD8 T-cell immune memory in syngeneic mouse models of glioma [119]. Delta24-RGDOX is currently being tested in clinical trials (see above).

Another co-stimulatory receptor is glucocorticoid-induced TNFR family-related gene (GITR). Stimulation with GITR-ligand (GITRL) leads to activation and proliferation of antigen-primed CD4 and CD8 T cells. Glioma treatment with the GITRL-armed oAd Delta24-GREAT resulted in expansion and activation of T cells with a high frequency of central memory CD8 T cells [120].

### 5.7. Arming with T Cell Engager Proteins

A promising strategy to redirect T cells to cancer cells are bispecific T-cell engagers (BiTEs) [121]. BiTEs are composed of a tumor ligand and a single-chain antibody fragment, which facilitates binding to CD3. BiTE-mediated clustering of tumor cells and T cells leads to T-cell activation and antitumor cytotoxicity thereby circumventing T-cell receptor (TCR)-mediated antigen recognition. However, the side effects of BiTEs can be considerable and therapeutic success in solid tumors has been rather limited. OVs armed with BiTEs can warrant high BiTE levels in tumor tissue and thus optimizes the ratio of on-target/off-tumor toxicity. Furthermore, viral tumor inflammation promotes intratumoral T-cell infiltration and thus provides an appropriate T-cell pool for BiTE-mediated T-cell retargeting. Fajardo et al. engineered an oAd to express an EGFR-BiTE (ICOVIR-15K-cBiTE) [122]. After expression in cancer cells, the BiTEs activated T cells in PBMCs in vitro. In murine xenograft models, ICOVIR-15K-cBiTE supported tumor infiltration and persistence of adoptively transferred T cells. ICOVIR-15K-cBiTE was also employed to overcome the limits of a CAR T-cell therapy because the EGFR-BiTE was able to redirect T cells against tumor cells that had lost the recognition antigen of the CAR [123]. Freedman et al. constructed a variant of enadenotucirev (EnAd) expressing a BiTE which targets epithelial cell adhesion molecule (EpCAM) [124]. The authors showed that crosslinking of EpCAM-expressing target cells and PBMC-derived T cells activated both CD8 and CD4 T cells. Furthermore, T cells in ascites fluid from cancer patients were activated by the virus-encoded BiTE and EpCAM-positive tumor cells were successfully depleted.

The use of BiTEs targeting components of the tumor microenvironment is a promising approach to reverse tumor immunosuppression. Freedman et al. modified EnAd to express a fibroblast activation protein (FAP)-targeting BiTE to redirect T cells to cancer-associated fibroblasts (CAFs) [125]. Treatment of biopsies of ascites or solid prostate cancer tissue samples with FAP-BiTE- expressing variant of EnAd was capable of activating tumor-infiltrating PD1^+^ T cells to kill CAFs. This in turn interfered with CAF-associated immunosuppression and resulted in an upregulation of proinflammatory cytokines, increased the presentation of tumor antigen, and finally led to improved T cell function. A comparable strategy has been pursued by generating the oAd ICO15K-FBiTE [126]. In a xenograft model, the expression of FBiTE led to an increased intratumoral T-cell accumulation and decreased the intratumor levels of FAP.

Tumor-associated macrophages (TAMs), and particularly the M2-polarized subset, contribute to immunosuppression. To deplete cancer-promoting TAMs and to reverse immunosuppression, Scott et al. recently developed EnAd-variants equipped with bi- and trivalent T-cell engagers targeting CD206 or folate receptor β on M2-like macrophages [127]. By detecting selective T-cell cytotoxicity against M2-TAMs in cancer patient biopsies, they could demonstrate that these BiTEs allow selective depletion of tumor-promoting TAMs whilst sparing those with potential antitumor features.

## 6. Oncolytic Adenoviruses in Multi-Stage Immunotherapies

During carcinogenesis, tumors use a wide variety of mechanisms to escape immunosurveillance, which may explain the heterogenous responses to checkpoint inhibitors. To make more tumors sensitive for this therapy, multi-stage immunotherapies are required in the future that address T-cell paucity and immunosuppression in the tumor from several sides. These multi-stage immunotherapies may include a virotherapy part for initial immunoactivation, an external support with tumor-directed T cells, and a systemic checkpoint intervention to maintain T cell activity. First steps towards multi-stage therapies are the current investigations on the synergy of OVs with checkpoint inhibitors. T-Vec and pembrolizumab have yielded encouraging interim results and data on long-term survival are eagerly awaited [7]. Corresponding studies with oAds are ongoing. Results from experimental models support this perspective. It has been shown after infection of B16 melanoma with Newcastle disease virus (NDV) that localized virotherapy and systemic CTLA-4 blockade led to rejection of infected and distant/non-infected tumors [128]. Using an oncolytic adenovirus, we demonstrated that intratumoral application sensitized CMT64 tumors for a systemic PD-1 antibody resulting in epitope spreading of neoantigen-specific T cells [129].

As an initiation step in multi-stage immunotherapies, virotherapeutic vectors must provide a solid basis for follow-up interventions. An important aim is to increase the immunogenicity of the used oAds e.g., by including additional danger signals such as CpG motifs [30]. Furthermore, oAds need to stimulate immune cells that augment direct cytotoxicity of oncolytic viruses or which support the shaping of optimal antitumor immune responses. It has been shown that contact dependent stimulation of NK cells can augment the therapeutic potential of oncolytic adenoviruses [130]. Based on the paradigm that effective CD8 T cell responses require the help of CD4 T cells, the Cerullo group has recently reengaged a pathogen-related CD4 T cell response to support an antitumor vaccination using peptide-loaded oAds (PeptiCRAds). In mice that had been preimmunized with tetanus, CD8 dependent immune responses, elicited with the oncolytic vaccine, were more effective when the used oAds were additionally loaded with a CD4-restricted tetanus peptide [131].

**Figure 1:**
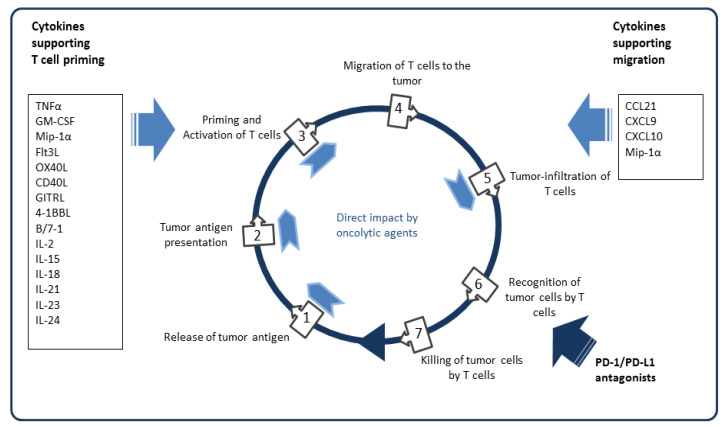
Immunostimulatory transgenes currently used in oncolytic adenoviruses in the context of the cancer immunity cycle.

Based on early experiences, repetitive dosage of oncolytic viruses has been regarded as mandatory to achieve a sufficient extent of tumor lysis. However, such a procedure may not necessarily yield the most effective anti-tumor immune responses. Robust anti-adenovirus responses may interfere with the activity as an in-situ vaccine. In the STEP-trial, an adenovirus-based vaccine against human immunodeficiency virus (HIV) was not capable of preventing HIV-infection. Instead, vaccine-treated men showed an even increased infection risk compared to control patients [132]. Studies with adenoviral vaccines in mice have confirmed that strong adenovirus epitopes may cause unresponsiveness to the vaccine [133]. Considering the prime/boost characteristics of repetitive oAd application, strong virus-derived antigens may outcompete the supposedly weaker tumor-associated antigens. Heterologous use of OVs is a promising approach to prevent the dominance of virus-specific immune responses. Application of an oAd followed by an oncolytic vaccinia virus eradicated established tumors in Syrian hamsters predominantly via strong tumor-specific T-cell immune response [134]. Interestingly, Tysome et al. found that this specific sequence was superior compared with the reverse combination, suggesting that viruses are differentially qualified for prime or boost, respectively. As an example for such a coordinated virus choice, it has been demonstrated in a murine B16 model that reovirus for triggering a CD8 Th1-dominated immune response can be combined with a subsequent CD4 Th17 helper response by vesicular stomatitis virus to achieve a potent T-cell pool for PD-1 inhibition [135]. Adenoviruses induce strong CD8 effector memory responses with a rather moderate potential for further expansion [136,137] and are therefore probably better suited to amplify an immune response initiated by alternative OVs. Heterologous administration also provides an option to select vectors with immunostimulatory arming adapted to specific needs of tumor immune activation. Whereas initial OV applications need to optimize antigen presentation and T-cell priming, subsequent applications need to promote T-cell migration and tumor infiltration (Figure 1).

OAds cause strong humoral immune responses. These neutralizing antibodies have been mostly regarded as an undesired adverse event in virotherapy that severely reduces virus efficacy and applicability. However, it has been demonstrated that fully neutralized OVs can still exert antitumoral effects through delivery in monocytes [138]. Moreover, it has been recently shown that preexisting immune responses can even improve the immune effect of oncolytic viruses [139], suggesting that neutralizing antibodies represent a so far unharnessed immune potential. We have recently described a strategy using bispecific adapter molecules to retarget adenovirus-neutralizing antibodies against tumor cells. This approach led to NK-cell dependent triggering of antitumor CD8 T cells and thus converted a limiting factor of virotherapy into an immunotherapeutic tool [140]. Tumor retargeting of antibodies could be a further option to fully exploit virotherapy-mediated tumor immune activation in multi-stage immunotherapies.

Regarding future multi-stage immunotherapies, oAds are promising tools for immunoactivation of solid tumors to facilitate adoptive cell therapy including CAR T cells. Tähtinen et al. have shown that tumor infection by oAds attracted leukocytes to the tumor, which promoted the intratumoral activity of adoptively transferred OT-1 T cells [141]. Watanabe and colleagues have demonstrated that the oAd TILT-123 improved the outcome of mesothelin-directed CAR-T cell therapy in models of pancreatic adenocarcinoma (PDAC) [87]. Intratumoral virotherapy with TILT-123 supported tumor infiltration with T cells expressing a mesothelin-targeted CAR and significant tumor regression could be confirmed. The authors have shown increased M1-polarization of macrophages and dendritic cell maturation, indicating that this combined therapy is able to overcome the highly immunosuppressive tumor microenvironment of this tumor entity.

When oAds are used for expression of immunostimulatory transgenes, immune modulation ends with the termination of oAd infection. Porter et al. developed an interesting strategy to uncouple the cytokine activity from the limitations of oAd infection by using helper-dependent adenoviruses (hdAd) [142]. The oAd, added to hdAds at a low ratio, provides the factors in trans that are required for efficient virus replication and spreading of helper-dependent vectors. This in turn warrants a prolonged expression of immunomodulators beyond the elimination of the oAd through the host’s immune response. A further advantage is the huge capacity of the helper-dependent viruses and the option to incorporate various effector genes to realize a multi-stage immunotherapy. The authors have generated a hdAd expressing an antitumor BiTE (against CD44v6), IL-12, and an anti-PD-1L minibody. This immunostimulatory array allowed additionally transferred CAR-T cells to control tumor growth in xenograft models including an orthotopic model of HNSCC. In summary, combinations of oncolytic Ads, hdAds, checkpoint inhibition, and autologous CAR T cells are a strategy with significant regulatory and technical challenges but with unparalleled clinical potential for cancer immunotherapy.

## 7. Conclusions

Oncolytic viruses have shown a tolerable safety profile in cancer immunotherapy. Current oncolytic viruses have demonstrated therapeutic efficacy but also limitations when applied as a monotherapy. Nevertheless, oncolytic viruses have an outstanding potential to immunoactivate tumors that are unresponsive to systemic immunotherapies. To convert these tumors into an immunoactivated state that is more likely to respond to systemic checkpoint inhibitor application, oAds are well suited. oAds are established and highly versatile platforms for the local delivery of immunoactivating factors to modulate intratumoral immune cell contexture and to break immune suppression. oAd-based strategies that address tumor-specific immune dysfunction by employing variable immune modifiers or by delivering complex arrays of immune stimulatory factors show great promise as an essential part of multi-stage immunotherapies.

## Figures and Tables

**Table 1:** Currently running clinical trials on oncolytic adenoviruses according to https://clinicaltrials.gov.

Agent/Virus Name	Virus Type	Trial No.	Status/Start Date	Indication	Admin.	Phase	Co-Therapy	Arming
**Phase I**								
BM-hMSCs- DNX-2401	Ad5-delta24-RGD (MSCs as carriers)	NCT03896568	Recr. 02/2019	Recurrent glioma	i.a.	I	Surgery	none
DNX-2401	Ad5-delta24-RGD	NCT03178032	Active, not recr. 05/2017	Brainstem glioma DIPG	local	I	Radiotherapy, chemotherapy	none
DNX-2440	Ad5-delta24-RGD-OX40L	NCT03714334	Recr. 10/2018	Glioblastoma	i.t.	I	-	OX40L
CAdVec	Binary oAd: Onc.Ad + helper-dependent (HD)-Ad	NCT03740256	Active, not yet recr. 09/2020	Diverse HER2 positive solid tumors	i.t.	I	HER2-specific autol. CAR T cells	not disclosed
Enadenotucirev/ Colo-Ad1	Ad3/11 Chimera	NCT03916510	Recr. 07/2019	Locally adv. rectal cancer		I	Chemoradiation	none
NG-641	Ad3/11 Chimera	NCT04053283	Recr. 01/2020	Adv./Metastatic epithelial tumors	i.t., i.v.	I	Chemotherapy, checkpoint inhibitors	FAP/CD3, CXCL9, CXCL10, IFNα
NG-350A	Ad3/11 Chimera	NCT03852511	Recr. 02/2019	Adv./Metastatic epithelial tumors	i.t., i.v.	I	-	Anti-CD40 Ab
VCN-01	Ad-DM-E2F-K-Δ24RGD- PH20	NCT03284268	Recr. 09/2017	Refractory retinoblastoma	intravitreal	I	-	Hyaluronidase
VCN-01	Ad-DM-E2F-K-Δ24RGD- PH20	NCT03799744	Recr. 05/2019	Squamous cell carcinoma of head and neck	i.v.	I	Durvalumab	Hyaluronidase
VCN-01	Ad-DM-E2F-K-Δ24RGD- PH20	NCT02045602	Active, not recr. 01/2014	Adv. solid tumors PDAC	i.v.	I	Gemcitabine, Abraxane	Hyaluronidase
Ad5-yCD/mutTKSR39rep-hIL12	Ad5-yCD/mutTKSR39rep- hIL12	NCT02555397	Unknown 08/2015	Prostate cancer	intra prostatic	I	-	yCD/mutTk/IL-12
Ad5-yCD/mutTKSR39rep-hIL12	Ad5-yCD/mutTKSR39rep- hIL12	NCT03281382	Recr. 07/2017	Metastatic PDAC	i.t.	I	5-FC, chemotherapy	yCD/mutTk/IL-12
OBP-301	Ad5-hTert-E1A-IRES-E1B	NCT02293850	Recr. 10/2014	HCC	i.t.	I	-	none
ONCOS-102	Ad5/3-D24-GMCSF	NCT03003676	Active, not recr. 12/2016	Advanced or unresectable melanoma	i.t.	I	Cyclophosphamide, pembrolizumab	GM-CSF
TILT 123	Ad5/3-D24-TNFα-IRES-IL2	NCT04217473	Recr. 02/2020	Metastatic melanoma		I	TIL	TNFα, IL2
**Phase I/II**								
ONCOS-102	Ad5/3-D24-GMCSF	NCT02963831	Recr. 09/2017	Colorectal, chemoresistant ovarian, appendiceal cancer	i.p.	I/II	Durvalumab	GM-CSF
ONCOS-102	Ad5/3-D24-GMCSF	NCT03514836	Recr. 05/2018	Castration-resistant advanced metastatic prostate cancer	i.t.	I/II	DCVAC/PCa	GM-CSF
ONCOS-102	Ad5/3-D24-GMCSF	NCT02879669	Active, not recr. 06/2016	Unresectable malignant pleural mesothelioma		I/II	Carboplatin, cyclophosphamide	GM-CSF
LOAd-703	Ad5/35	NCT04123470	Recr. 01/2020	Malignant melanoma	i.t.	I/II	Atezolizumab	CD40L, 4-1BBL
LOAd-703	Ad5/35	NCT03225989	Recr. 03/2018	PDAC/ovarian, biliary, colorectal cancer	i.t.	I/II	Standard chemotherapy or Gemcitabine	CD40L, 4-1BBL
LOAd-703	Ad5/35	NCT02705196	Recr. 11/2016	PDAC	i.t.	I/II	Gemcitabine, Nab-Paclitaxel, atezolizumab	CD40L, 4-1BBL
AdVince	Ad5(PTD)CgA-E1AmiR122	NCT02749331	Recr. 03/2016	Neuroendocrine tumors	i.a.	I/II	-	none
ORCA-010	Ad5-Δ24RGD; T1-mut.	NCT04097002	Recr. 11/2019	Prostate cancer	i.t.	I/II	-	none
**Phase II**								
ADV/HSV-tk	Ad5	NCT03004183	Recr. 07/2017	Metastatic NSCLC TNBC	i.t.	II	Valacyclovir, SBRT radiation, pembrolizumab	HSV-tk
DNX-2401	Ad5-delta24-RGD	NCT02798406	Active, not recr. 06/2016	Brain cancer	i.t.	II	Pembrolizumab	none
OBP-301	Ad5-hTert-E1A-IRES-E1B	NCT03190824	Active, not recr. 12/2016	Melanoma stage III|stage IV	i.t.	II	-	none
OBP-301	Ad5-hTert-E1A-IRES-E1B	NCT03921021	Recr. 05/2019	Esophagogastric adenocarcinoma	i.t.	II	Pembrolizumab	none
CG0070	Ad-E2F-E1A-E3-GM- CSF	NCT04387461	Not yet recr. 08/2015	NMIBC	intra vesical	II	Pembrolizumab	GM-CSF
**Phase III**								
CG0070	Ad-E2F-E1A-E3-GM- CSF	NCT04452591	Not yet recr. 09/2020	NMIBC	intra vesical	III	N-dodecyl-B-D-maltoside	GM-CSF
H101	Ad5	NCT03780049	Recr. 10/2018	Non-resectable HCC	i.a.	III	HAIC 5-FU, leucovorin	none

Abbreviations: i.a., intraarterial; i.v., intravenous; i.t., intratumoral; i.p., intraperitoneal; HCC, hepatocellular carcinoma; NMIBC, non-muscular invasive bladder cancer; NSCLC, non-small cell lung cancer; TNBC, triple negative breast cancer, PDAC, pancreatic adenocarcinoma; DIPG, diffuse intrinsic pontine glioma; GM-CSF, granulocyte-macrophage stimulating factor; 5-FU, 5-fluorouracil; 5-FC, 5-fluorocytosine; HAIC, hepatic artery infusion chemotherapy; SBRT, stereotactic body radiation therapy; hMSC, human mesenchymal stem cells; DCVAC/PCa, autologous dendritic cells pulsed with killed LNCaP prostate cancer cells; TIL, tumor-infiltrating lymphocytes; TNFα, tumor necrosis factor alpha; IL2, interleukin 2; IL-12, interleukin 12; HSV-tk, herpes simplex thymidine kinase; CXCL, chemokine ligand; FAP, fibroblast activation protein; IFNα, interferon alpha; CAR, chimeric antigen receptor.
